# Element-specific detectors for high pressure liquid chromatography (HPLC): a computerized, automated HPLC-graphite furnace atomic absorption system

**DOI:** 10.1155/S1463924687000026

**Published:** 1987

**Authors:** S. J. Haswell, R. A. Stockton, K. C. C. Bancroft, P. O'Neill, A. Rahman, K. J. Irgolic

**Affiliations:** 1 John Graymore Chemical Laboratories Department of Environmental Sciences Plymouth Polytechnic Drake Circus Devon Plymouth UK; 2 Chemistry Department Texas A & M University College Station Texas 77843 USA


Journal of Automatic Chemistry/*Journal of Clinical Laboratory Automation*, Vol. 9, No. 1 (January–March 1987), pp. 6–14

### Element-specific detectors for high pressure liquid chromatography (HPLC): a computerized, automated HPLC-graphite furnace atomic absorption system

**S. J. Haswell[1], R. A. Stockton[2], K. C. C. Bancroft[1], P. O'Neill[1], A. Rahman[1] and K. J. Irgolic[2]***

[1] *John Graymore Chemical Laboratories, Department of Environmental Sciences Plymouth Polytechnic, Drake Circus, Plymouth, Devon, UK*
[2] *Chemistry Department, Texas A & M University, College Station, Texas 77843, USA*

### Introduction

Liquid chromatography, and especially high pressure liquid chromatography, with the great resolving power of its microparticulate column materials are potentially the best techniques for the separation and determination of trace substances that have insufficient volatility for analysis by gas chromatography. Many of these trace substances contain a metal or a metalloid such as arsenic, selenium, tin or cobalt, that can be determined by atomic absorption spectrometry. These compounds may be essential for life [1], for example cobalamine, or pose a threat to organisms [2], for example arsenite. For environmental and health reasons, knowledge about the nature and concentrations of such substances in living and non-living matter is critical. These substances occur at mg/l or µg/l concentrations in complex matrices which will obscure chromatographic peaks of interest or make their identification difficult. To overcome these difficulties graphite furnace atomic absorption spectrometers [3–10] and plasma emission spectrometers [11 and 12] have been used as element-specific detectors for high pressure liquid chromatography in manual [4] and automated modes [3, 5, 8 and 12].

This paper describes the construction and use of a computerized, automated interface between a high pressure liquid chromatograph (HPLC) and an Instrumentation Laboratory Model IL-555/151 graphite furnace atomic absorption spectrometer (GFAAS).

### Construction of the HPLC-GFAAS system

The HPLC-GFAAS system was assembled from the following components: a Waters Associates Model 6000A pump; a Beckman high pressure sample injection valve; a Hypersil column (25 cm × 0·4 cm, 3–5 µm silica gel coated with octadecylsilyl groups); an Instrumentation Laboratory Model IL-555/151 graphite furnace atomic absorption spectrometer; two eight-port slider injection valves with pneumatic actuators; eight solenoid valves; an injector for the deposition of samples into the graphite cuvette; various electronic components (table 1) that comprise the electronic interface between the HPLC and the GFAAS; an Ohio Scientific Inc. Superboard II, single-board computer with a 6502 microprocessor, 8 kilobytes of RAM, and an 8 kilobyte BASIC in ROM; an audio cassette recorder for mass storage of programs and data; and a 12-in black and white television made for the USA market to serve as the monitor. A 60-cycle pulse train was obtained from the verticle blanking for the television and connected to the non-maskable interrupt pin on the CPU of the computer through a single-pole, single-throw switch (timing switch) mounted on the front panel. A diagram of the HPLC-GFAAS system is shown in figure 1.

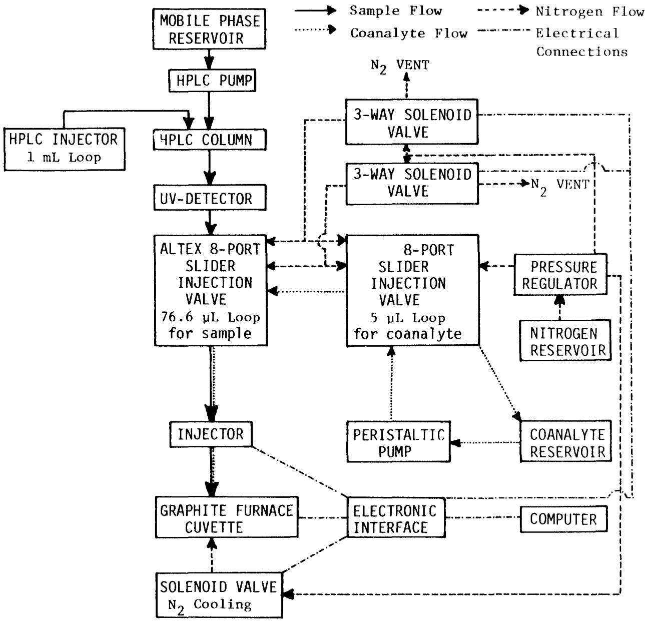

*Figure 1. Block diagram for the HPLC-GFAAS interface.*

The exit of the HPLC column was connected to the UV detector with 1·5 mm OD stainless-steel tubing. All other lines transporting liquid and the pressure relief loop were cut from Teflon tubing (1·5 mm OD, 0·3 mm ID) and were kept as short as possible. Teflon tubing of approximately 3·1 mm OD, 2·4 mm ID served as nitrogen lines. Flanged Nylon fittings were used as connectors. The loops for the Beckman sampling valve (76 µl) and the Alltech coanalyte valve (5 µl) were cut from 1·5 mm OD, 0·8 mm ID Teflon tubing. The position of these two

* Author to whom correspondence should be addressed.

valves in the HPLC-GFAAS system is show in figure 1. The flow of sample and coanalyte through the valves is discussed in the section on 'Operation of the HPLC-GFAAS system'.

The sample coming from the sampling valve was delivered into the graphite cuvette by the injector (figure 2). The injector is assembled from a solenoid, a return spring, a stainless-steel rod, and a stainless-steel hypodermic needle. An axial hole (1·5 mm diameter) was drilled into the rod to a depth of 25 mm. Another hole was drilled into the rod from the side to meet the end of the axial hole at an angle of approximately 150°. The Teflon tubing (1·5 mm) that delivers the sample from the sampling valve to the graphite cuvette was pushed through the side hole and the axial hole until it emerged from the rod. A hypodermic needle was inserted into the Teflon tubing, which was then drawn into the axial hole until a tight fit was obtained. This rod assembly was then screwed into the back of the solenoid plunger. The solenoid assembly was mounted on an aluminium plate that provided a stop for the plunger. The return spring was attached to the plunger and the bent-up end of the aluminium plate (figure 2). This plate was fixed to a sturdy aluminium base plate via three adjustable bolts that allowed the height of the injection needle to be adjusted. The base plate was screwed to the spectrometer housing. The length of the needle was adjusted by pushing it into the axial hole in the stainless-steel rod. The microswitch was mounted on the adjustable aluminium plate. The microswitch lever was positioned to drop into the furnace cavity and close the switch when the furnace door opens by swinging into the furnace cavity.

The upper port to the furnace was plugged. A stainless-steel tube (1·5 mm OD) was inserted into the hole drilled through the centre plug. This tube was directed toward the graphite cuvette for delivery of nitrogen as a coolant.

The components for the electronic interface and the required circuits were placed on a 12 × 12 in board. On-board connections were made with wire wrapping techniques. Off-board connections were terminated on barrier blocks. Only the computer and the interface

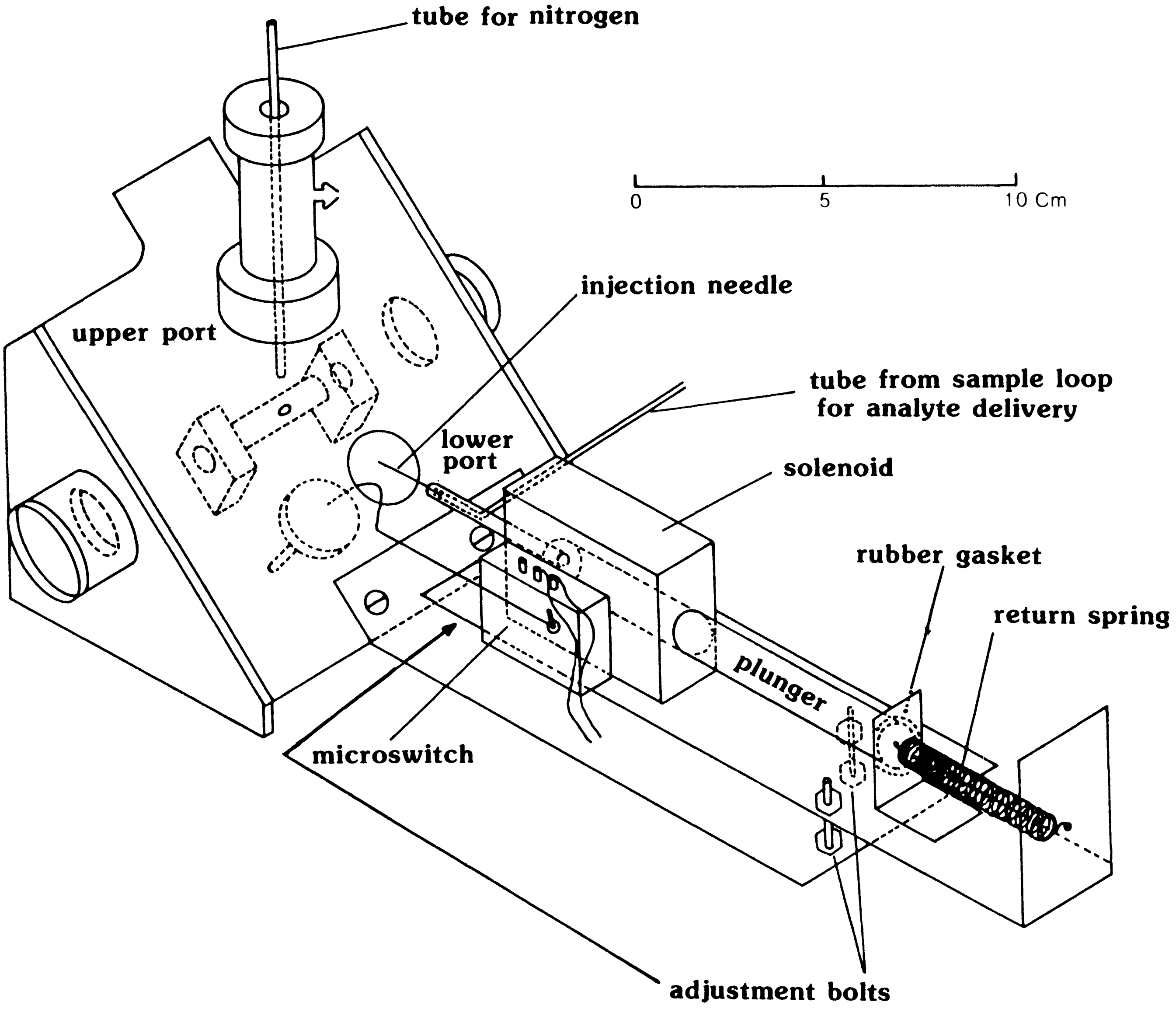

*Figure 2. Injection system.*

circuits were connected by dip connectors. The electronic diagrams for the address buffering and decoding logic, data buffering circuits, interface controller, analogue selection and conditioning, and the analogue-to-digital conversion are shown in figure 3.

*Table 1. Components for the construction of the HPLC-GFAAS interface.*

| | |
|---|---|
| ELECTRONIC COMPONENTS | |
| Amplifier, operational, 741 | 3 |
| Analogue Switch, 4502 CMOS | 1 |
| Buffer, 74LS245 | 4 |
| Buffer, 7406 | 3 |
| Buffer, 4049 CMOS | 2 |
| Capacitor, electrolytic, 10 μF | 1 |
| Capacitor, electrolytic, 100 μF | 1 |
| Capacitor, tantalum, 10 μF | 1 |
| Converter, A/D, 8-bit, ADC0800 | 1 |
| Counter, 4-bit, 74LS160 | 1 |
| Decoder, 1 to 16, 74LS154 | 1 |
| Diode, Zener, 9·0 V, 1 W | 1 |
| Diode, Zener, 9·4 V, 1 W | 2 |
| Diode, Signal, 1N914 | 6 |
| 3-Input AND Gate, 74LS11 | 1 |
| 8-Input NAND Gate, 74LS30 | 1 |
| Inverter, 74LS04 | 1 |
| Microswitch, normally open | 1 |
| Peripheral Interface Adapter, 6521 | 2 |
| Potentiometer, 1 kiloohm, 0·25 W | 2 |
| Potentiometer, 10 kiloohm, 0·25 W | 2 |
| Relay, DPST, <30 V, <40 mA | 1 |
| Relay, SPST, <30 V, <40 mA | 5 |
| Resistor, 0·1 kiloohm, 0·25 W | 2 |
| Resistor, 1·0 kiloohm, 0·25 W | 1 |
| Resistor, 2·2 kiloohm, 0·25 W | 9 |
| Resistor, 6·2 kiloohm, 1%, 0·25 W | 1 |
| Resistor, 10·0 kiloohm, 1%, 0·25 W | 1 |
| Resistor, 15·0 kiloohm, 1%, 0·25 W | 1 |
| Resistor, 20·0 kiloohm, 1%, 0·25 W | 4 |
| Resistor, 99·9 kiloohm, 1%, 0·25 W | 2 |
| Transistor, NPN, 2N222 | 1 |
| COMPUTER SYSTEM | |
| Audio Cassette Recorder | 1 |
| Computer, single board, 6502 microprocessor, 8 kilobytes RAM, 8 kilobyte BASIC in ROM, Ohio Scientific | 1 |
| Television, 12-inch, black & white (US market) | 1 |
| SAMPLING SYSTEM | |
| Coupling, Nylon, Beckman | 10 |
| Fittings, flanged, Nylon for 1·5 mm and 3·1 mm tubing, Beckman | 20 |
| Slider Injection Valve, 8-port with pneumatic actuator, Beckman | 2 |
| Solenoid Valve, 3-Way, 220 V | 2 |
| Tubing, Teflon 1·5 mm OD × 0·3 mm ID | 10 feet |
| 1·5 mm OD × 0·8 mm ID | 1 foot |
| 3·1 mm OD × 2·4 mm ID | 10 feet |
| Tubing, stainless steel, 1·5 mm OD | 5 feet |
| INJECTION SYSTEM | |
| Aluminium Plate, 22 × 3 × 0·5 cm, 15 × 6 × 0·5 cm, 4 × 2 × 0·2 cm | 1 each |
| Needle, hypodermic, stainless steel | 1 |
| Rod, stainless steel | 1 |
| Solenoid, 220 V | 1 |
| Solenoid Valve, 2-Way, normally closed, 220 V | 1 |
| Spring, return | 1 |

### Operation of the HPLC-GFAAS system

The liquid chromatograph delivers a continuous stream of analyte. The GFAA spectrometer, however, operates discontinuously. Each aliquot of the column effluent injected into the graphite cuvette must be evaporated to dryness during the drying cycle, the organic material in the residue must be mineralized during the ashing cycle, and the inorganic material finally atomized at high temperatures. Before the next aliquot can be introduced, the cuvette must be cooled. The discontinuous operation of the spectrometer demands that aliquots of the effluents are taken from the effluent stream at appropriate intervals determined by the cycling time of the spectrometer. This operation is performed by the sampling subsystem, the major parts of which are two eight-port slider injection valves. The aliquots of the effluent and coanalyte are routed from the sampling and coanalyte valves to the injection device that deposits the aliquots into the graphite cuvette via a delivery needle. The correct sequence of events is controlled by the electronic interface and the computer. The following events must occur in the order given, after the slider injection valves have been switched to allow the effluent and coanalyte to flow through the respective sample loops:

Insertion of the delivery needle into the graphite cuvette.
Switching of the sampling and coanalyte valves to the inject position.
Transport of the sample and coanalyte into the cuvette.
Switching of the sampling and coanalyte valves to the standby positions.
Retraction of the delivery needle from the cuvette.
Initiation and completion of the GFAAS analysis cycle (drying, ashing, atomization).
Transmittance of absorption data collected during the atomization cycle via the electronic interface to the computer for print-out and storage.
Monitoring of the cuvette temperature.
Opening of the furnace door when the cuvette temperature has dropped to 750 °C.
Activation of the solenoid valve for an appropriate period to allow a stream of nitrogen to cool the graphite cuvette.
Return to event 1 and repetition of the sequence of events after the cuvette temperature has decreased to a predetermined level (120–170 °C depending on the element to be determined).

### *Sampling*

The major parts of the sampling device are the sampling and coanalyte valves. These valves have a load and an inject position and are switched simultaneously. Aliquots are removed from the continuous stream of the effluent coming from the HPLC column by the sampling valve, an eight-port slider injection valve (figure 4). Two pairs of ports (1, 2 and 3, 4) are connected by short pieces of 0·8 mm ID Teflon tubing to form a sample loop (ports 1 and 2) and a bypass loop (ports 3 and 4). One of the eight ports (port 8) is closed in the load position. The line coming from port 5 of the coanalyte valve providing nitrogen and coanalyte is connected to port 8. The

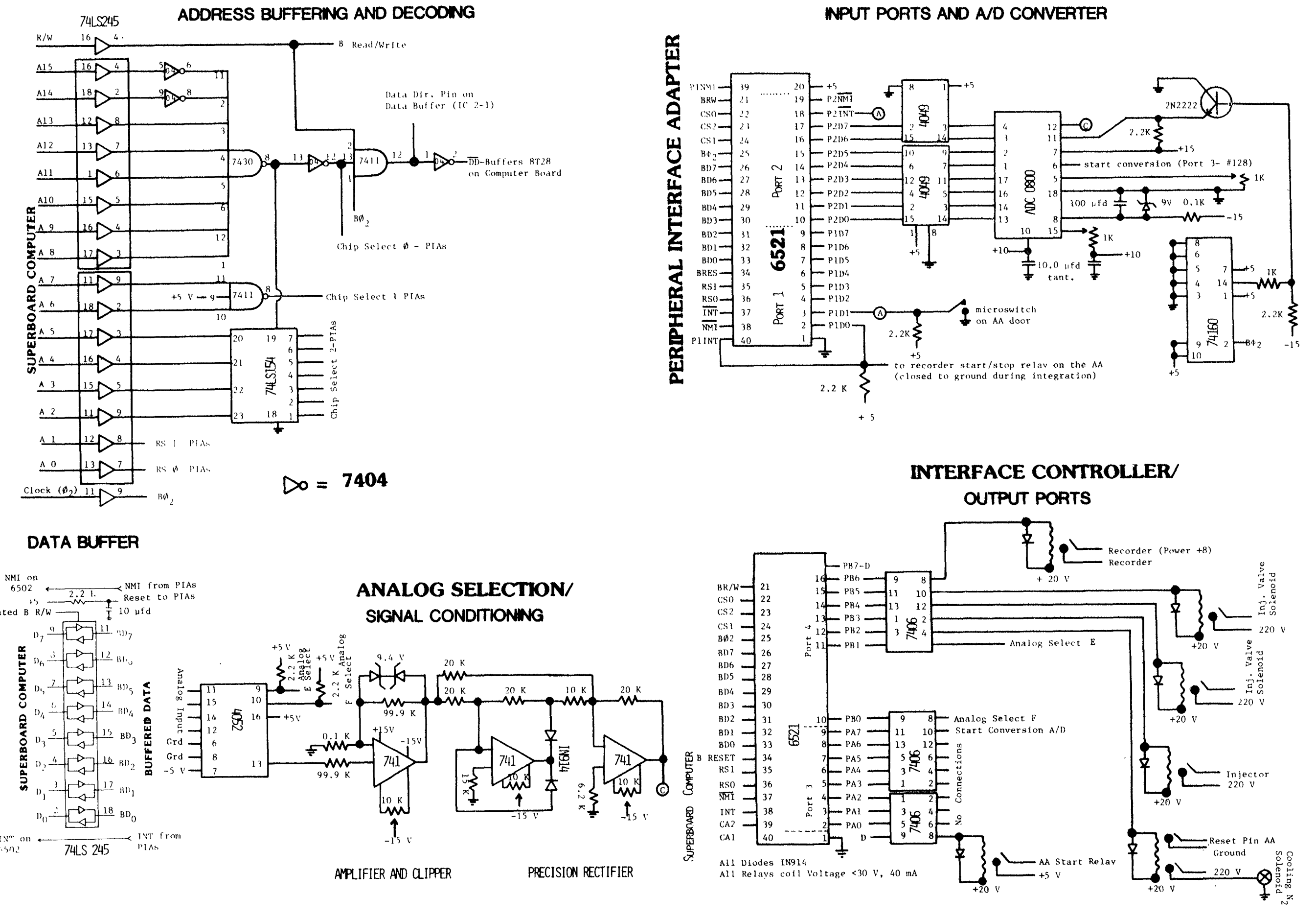

*Figure 3. Diagrams for the electronic part of the HPLC-GFAAS interface.*

connections to the other ports are made as shown in figure 4. In the standby position the effluent flows from the column to port 6 of the sampling valve, through the sample loop and port 7 to the fraction collector or to waste. In the inject position the effluent flows from the column to port 6, through the bypass loop and port 7 to the fraction collector or to waste. Port 8 is opened and linked via the sample loop to port 5, which leads to the injector. Nitrogen at 60 psi and an aliquot of the coanalyte coming from the coanalyte valve push the aliquot of the effluent isolated in the sample loop to the injector and into the graphite cuvette. When the sample and the coanalyte aliquots have been deposited in the cuvette, the valves are switched back into the standby position. During the switching the liquid flow is interrupted for a short period. To avoid build-up of pressure in the HPLC system and damage to the column and detectors, a three-foot pressure relief loop was installed that bypasses the sampling valve as shown in figure 4.

The coanalyte valve, another eight-port slider injection valve, makes it possible to add a coanalyte that enhances the sensitivity, minimizes interferences during the GFAAS analysis, and flushes the tubing leading to the graphite cuvette. Flushing of the tubing with the coanalyte assures better transfer of the sample and minimizes memory effects. The coanalyte valve has a coanalyte loop (ports 1, 2; 5 μl) and a bypass loop (ports 3, 4). A peristaltic pump delivers the coanalyte solution from the reservoir to port 6. In the standby position the coanalyte flows from port 6 through the coanalyte loop to port 7 back into the reservoir. Port 8, to which the nitrogen line is attached, is closed in the standby position. In the inject position the coanalyte flows through the bypass loop to port 7 and back into the reservoir. The nitrogen port (8) is connected to the coanalyte loop, and the coanalyte loop to port 5 that is linked to port 8 of the sampling valve. The nitrogen forces the aliquot of the coanalyte in the coanalyte loop of the coanalyte valve through port 5 to port 8 of the sampling valve. A pressure relief loop is not needed for the coanalyte valve. The determination to which ports the sample and bypass loops and the other lines must be connected can be made with information provided by the manufacturer or by experimentation that reveals the flow patterns for the two valve positions.

The sampling valve and the coanalyte are simultaneously switched by the two pneumatic actuators that are part of each valve. The actuators are brought into action by nitrogen pressure (60 psi) applied via two solenoid valves that are under computer control (see figure 1).

### *Injector*

The injector (see figure 2) transfers aliquots of the column effluent and coanalyte into the graphite cuvette. Just before the sampling and coanalyte valves are switched to the inject position, power is applied to the solenoid.

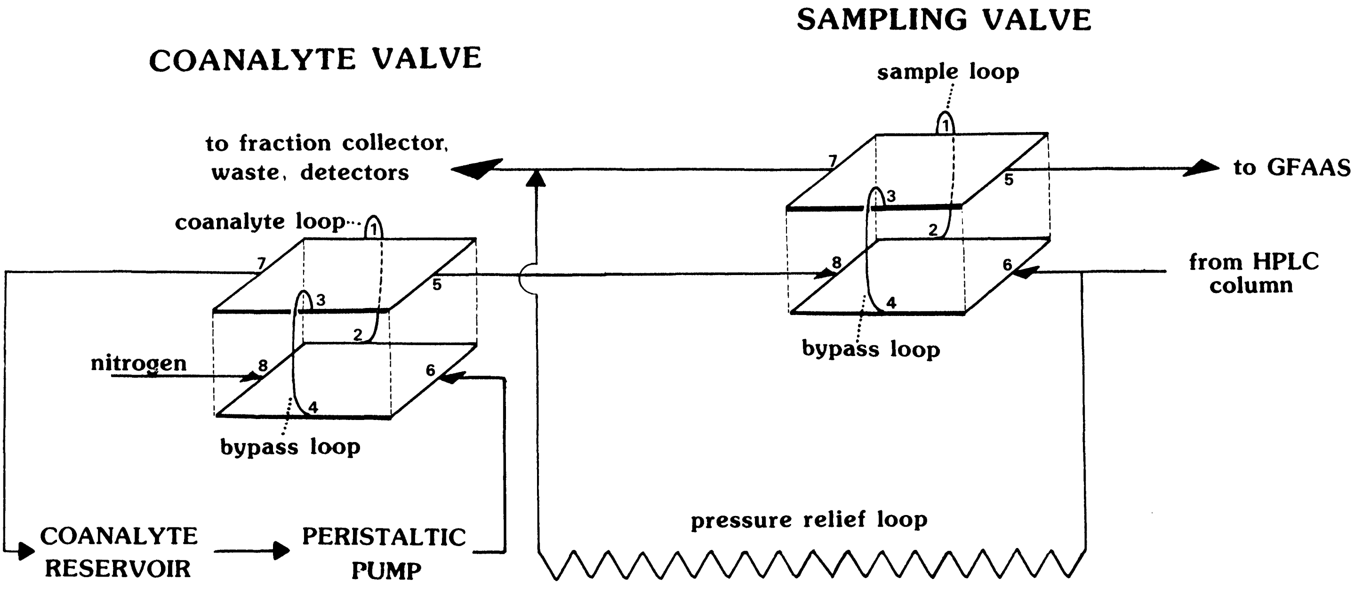

*Figure 4. The sampling system of the HPLC-GFAAS interface.*

Under these conditions the plunger is pulled into the solenoid and the injection needle is pushed through the lower, open port of the furnace chamber into the graphite cuvette. After the injection has been completed and before the drying cycle begins, power is removed from the solenoid. The spring pulls the plunger from the solenoid and the injection needle from the cuvette.

*Electronic interface*

The electronic interface performs two functions. First, it receives commands from the computer and translates these commands into actions required for the operation of the HPLC-GFAAS system. Secondly, it converts the analogue signals from the detectors (UV, GFAAS, thermocouple) into digital signals which the computer is capable of processing.

The address decoding logic is shown in figure 3. The port addresses are set by the IC chips 7404, 7430, 7411, and 74LS154. Port 1 is addressed at memory location 16384 (base 10). Each of the next 15 ports is three memory locations higher than the previous port because the setting of the two registers of each peripheral interface adapter require the lower two bits. The HPLC-GFAAS system described in this paper requires only four ports. The remaining ports are available for future expansion, for instance, for additional detectors. The data lines between the interface and the computer are buffered (figure 3) using a 74LS245 transceiver buffer properly gated by the buffered read/write signal from the address decoding logic. The maskable and nonmaskable interrupts between the interface and the computer are not buffered. The buffer output is connected to the 6521 peripheral interface adapter in the analogue-to-digital conversion circuit (figure 3).

The interface controller circuit (figure 3) controls the relays in the interface. The relays in turn control the power to the solenoid operating the injection needle, to the nitrogen solenoid valves providing 60 psi nitrogen to the pneumatic actuators in the sampling and coanalyte valves, to the solenoid valve supplying nitrogen to cool the cuvette, and to the GFAAS start and reset switches. These relays are activated by the appropriate bit pattern on the 6521 IC (port 4) which is buffered by the 7406s. Any relay that has a coil voltage less than 30 V and a coil current less than 40 milliamps may be used. Port 4 of this peripheral interface adapter controls the Analogue Select bits. These bits determine which analogue input will be read by the analogue-to-digital converter. Port 3 controls the relay which starts the GFAAS analysis sequence and also the 'start conversion sequence' for the analogue-to-digital converter.

The analogue input from any of four 10 V signals is converted by the analogue-to-digital conversion circuit (figure 3) to the digital data required by the computer. The analogue-to-digital converter (ADC 0800) is buffered from port 2 of the peripheral interface adapter by 4049 CMOS buffers. Both ports of this peripheral interface adapter are configured as inputs. Port 2 receives the 8 bits of data from the converter, whereas port 1 is used to signal to the computer that the GFAAS door is open and ready for another sample. This arrangement became necessary, because the IL-555 furnace door did not reliably open at a preset temperature. The time for the introduction of a new sample is now determined by the temperature read-out from the temperature sensor in the graphite furnace.

The analogue selection and signal conditioning circuit (figure 3) amplifies the outputs of the spectrometer, of the temperature read-out, and of any additional detectors ($\pm 10$ mV) by a factor of 1000 and rectifies the signals to a positive voltage required by the analogue-to-digital converter. The first 741 operational amplifier after the 4052 analogue selection chip amplifies the analogue input to a maximum $\pm 10$ V. The next two 741 operational amplifiers serve as a precision rectifier that converts any negative to positive voltage.

*Software*

The two programs necessary for the operation of the HPLC-GFAAS system are listed in tables 2 and 3. The assembly language program (table 2) required to determine the switch-time, increments a counter every time the non-maskable interrupt is activated by the 60-cycle pulse train. After every 60 cycles (1 s) the CRT display is updated by changing the appropriate memory location in the video memory space. The program starts incrementing the display when the computer is turned on and the timer switch on the computer front panel is on. The BASIC program (table 3) sets the appropriate memory locations and thus the switch-time to zero when the space bar and carriage return are depressed at the time the sample is injected. The BASIC program (table 3) controls the HPLC-GFAAS system. A line by line description of this program is given in table 4.

### Separation and determination of arsenic compounds

The operability of the HPLC-GFAAS system was checked with a solution containing arsenite, methylarsonic acid, and dimethylarsinic acid. The compounds were separated isocratically on a Hypersil ODS (3–5 μm) column with 0·1 M aqueous ammonium formate as the mobile phase.

The GFAAS was set up according to the manufacturer's specifications. The computer is turned on and the BASIC program (table 3) that is stored on a cassette is loaded. The timing switch that transmits the timing pulses to the CPU must be open. After the program is loaded, <CR>

*Table 2. 6502 Assembly language program for the determination of the switch time*.*

| | | | |
|---|---|---|---|
| 1 | *=$130 | 115 | CLC |
| 2 | COUNT = 255 | 120 | ADC #1 |
| 3 | DESC = 54170 | 125 | CMP #58 |
| 4 | DTSEC = DSEC-1 | 130 | BCS TENSEC |
| 5 | DHSEC = DESC-2 | 135 | STA DSEC |
| 6 | DTHSEC = DSEC-3 | 140 | JMP EXIT |
| 7 | TIMEL = 54147 | 200 | TENSEC STY DSEC |
| 8 | TIMEH = 54115 | 205 | LDA DTSEC |
| 15 | PHA | 210 | CLC |
| 20 | LDA COUNT | 212 | ADC #1 |
| 25 | CLC | 215 | CMP #58 |
| 30 | ADC #1 | 220 | BCS HUNSEC |
| 35 | CMP #60 | 225 | STA DTSEC |
| 40 | BCS SECOND | 230 | JMP EXIT |
| 45 | STA COUNT | 235 | HUNSEC STY DTSEC |
| 50 | PLA | 240 | CLC |
| 51 | RTI | 245 | LDA DHSEC |
| 55 | SECOND TXA | 250 | ADC #1 |
| 56 | PHA | 255 | CMP #58 |
| 57 | TYA | 260 | BCS THOSEC |
| 58 | PHA | 265 | STA DHSEC |
| 60 | LDX #0 | 270 | JMP EXIT |
| 65 | LDY #$30 | 275 | THOSEC STY DHSEC |
| 70 | STX COUNT | 280 | CLC |
| 75 | LDA TIMEL | 285 | LDA DTHSEC |
| 80 | CLC | 290 | ADC #1 |
| 85 | ADC #1 | 295 | STA DTHSEC |
| 90 | STA TIMEL | 1000 | EXIT PLA |
| 95 | LDA TIMEH | 1005 | TAY |
| 100 | ACD #0 | 1010 | PLA |
| 105 | STA TIMEH | 1015 | TAX |
| 110 | LDA DSEC | 1025 | PLA |
| 115 | CLC | 1030 | RTI |

* Switch-time: time elapsed between injection of the sample and switching of the sample and coanalyte valves into the inject position. This program increments memory locations in the video memory upon interrupt from the 60-cycle pulse train.

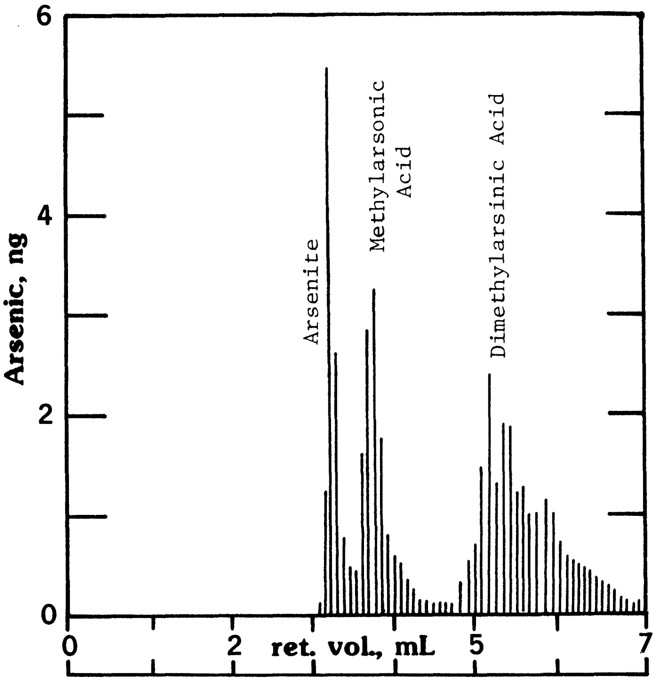

*Figure 5. Separation of arsenite, methylarsonic acid, and dimethylarsinic acid by the HPLC-GFAAS system.*

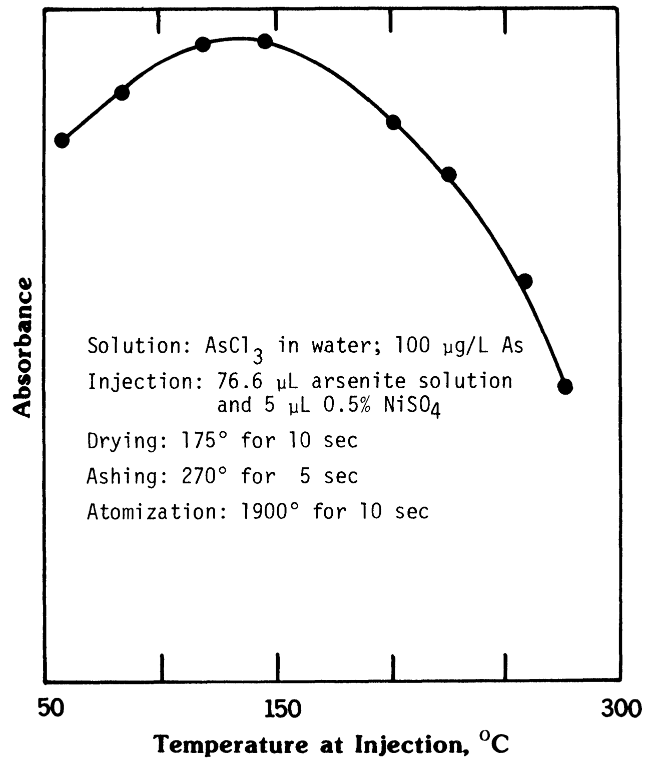

*Figure 6. The dependence of the absorbance on the temperature of the graphite cuvette at the time of the injection.*

is entered via the keyboard and the timing switch is closed. The injector leading to the chromatographic column is loaded and then placed into the inject position. At this time the space bar on the keyboard is depressed to start the operation of the HPLC-GFAAS system.

The chromatogram is shown in figure 5. For a quantitative determination of the arsenic compounds the signals defining the peak of a particular compound can be summed. These sums obtained for several concentrations of a compound can be used to construct a calibration curve [13].

The discontinuous operation of the GFAAS makes it possible that a compound eluting in a very narrow band might be missed by the detector. Therefore, it is desirable to make the time between analyses as short as feasible. The length of the period between analyses is determined by the time required for the drying/ashing/atomization cycle and the time needed to cool the cuvette to a temperature at which the next sample can be safely injected. The cooling time can be minimized by injecting the samples into the cuvette at the highest temperature suitable for the samples and by rapid cooling of the cuvette to this temperature by a stream of nitrogen. For

*Table 3. Program for the control of the interface in BASIC.*

```
    1 GOSUB 999:REM PORT SET-UP
    2 GOSUB 18090: REM SET-UP OF REAL TIME
      CLOCK
    3 PRINT:PRINT:PRINT"SWITCH ON
      TIMER":PRINT:PRINT
    7 PRINT "PRESS SPACE BAR THEN <CR> WHEN
      READY TO BEGIN"
    8 INPUT A$: IF A$ <>"" THEN 7
   10 DIM T(200),BACK(200),A(200),U(200)
   15 REM RESETTING SWITCH TIME CLOCK TO
      ZERO
   20 FOR I=54167 TO 54170:POKE I,48:NEXT
  199 REM WAITING FOR GFAA DOOR
  200 IF PEEK (16360) <>254 THEN 200
  201 Z=Z+1
  205 BACK = 0
  206 COUNT = 0
  208 REM DETERMINING BACKGROUND
  209 POKE 16366,0:REM ANALOG SELECT FOR
      GFAA
  210 POKE 16364,128:POKE 16366,0:REM START
      PULSE FOR A/D
  211 BACK = BACK + PEEK (16362)
  214 COUNT = COUNT + 1:IF COUNT <>5 THEN
      210
  215 BACK (Z) = BACK/5
  218 REM SWITCH TIME
  220 T(Z) = (PEEK(54167)-48)** 1000 + (PEEK (54168)
      - 48) * 100
  221 T(Z) = T(Z) + (PEEK(54169)-48) * 10 +
      (PEEK(54170) - 48)
  225 REM INJ. IN (8) AND SOL. OPEN (16)
  230 POKE 16366, 8 + 16
  240 FOR I =1 TO 1400:NEXT:REM DELAY FOR IN.
      VALVE TO CLOSE
  262 REM END GFAA START PULSE
  265 POKE 16366,0
  268 REM WAITING FOR INT. SIG. FROM GFAA
  270 IF PEEK (16360) <>253 THEN 270
  271 REM DATA ACQUISITION SUBROUTINE
  272 GOSUB 500
  273 POKE 16366,3:FOR I = 1 TO 770:NEXT:REM
      ANALOG SELECT FOR TEMP
  274 POKE 16364,128:POKE 16364,0
  275 IF PEEK (16362) > 230 THEN 274
  276 POKE 16366, 3 + 4:REM RESET GFAA AND
      COOLING NITROGEN ON
  279 REM LOWER TEMP SET-UP
  280 POKE 16364,128:POKE 16364,0:FLAG = FLAG + 1
  281 TEMP = TEMP + PEEK (16362)
  282 IF FLAG 5 THEN 280
  283 TEMP = TEMP/5
  284 REM CHANGE TEMP VALUE TO CHANGE
      INJECTION TEMPERATURE
  285 IF TEMP > 55 THEN FLAG =0:GOTO 280
  286 FLAG = 0
  287 TEMP = 0
  300 GOTO 200
  500 REM DATA ACQUISITION
  510 POKE 16366,0:REM ANALOG SELECT FOR
      GFAA
  520 POKE 16364,128:POKE 16364,0:REM START
      PULSA FOR A/D
  524 REM COUNTER FOR # DATA POINTS DURING
      INTEGRATION
  525 K = K + 1
  530 A(Z) = A(Z) + PEEK (16362)
  550 IF K < 100 THEN 520
  560 K = 0
  570 POKE 16366,1:REM ANALOG SELECTION OF
      U.V.
  580 POKE 16364,128:POKE 16364,0:REM A/D START
      CONVERSION PULSE
  590 U(Z) = PEEK (16362)
  599 POKE 517,255
  600 PRINT T(Z);A(Z);BACK(Z);A(Z) - (BACK*100)
  610 RETURN
  999 REM PORT SET-UP
 1000 POKE 16361,0:REM PORT 1
 1010 POKE 16360,0
 1020 POKE 16361,4
 1030 POKE 16363,0:REM PORT 2
 1040 POKE 16362,0
 1050 POKE 16363,4
 1060 POKE 16365,0:REM PORT 3
 1070 POKE 16364,225
 1080 POKE 16365,4
 1090 POKE 16367,0:REM PORT 4
 1100 POKE 16366,225
 1110 POKE 16367,4
 1120 RETURN
18080 REM REAL TIME CLOCK
18090 FOR I = 304 TO 416
18092 READ A
18094 POKE I,A
18096 NEXT I
18100 RETURN
19000 DATA 72,165,255,24,105,1,201,60,176,4,133
19001 DATA 255,104,64,138,72,152,72,162,0
19002 DATA 160,48,134,255
19003 DATA 173,131,211,24,105,1,141
19004 DATA 131,211,172,99,211,105,0,141,99,211
19005 DATA 173,154,211,24,105,1,201,58,176,6
19006 DATA 141,154,211,76,155,1,140,154,211,173
19007 DATA 153,211,24,105,1,201,58,176,6,141
19008 DATA 153,211,76,155,1,140,153,211,24,173
19009 DATA 152,211,105,1,201,58,176,6,141,152
19010 DATA 211,76,155,1,140,152,211,104,168,104,170
19011 DATA 211,105,1,141,151,211,104,168,104,170,104,64
```

*Table 4. Explanations for the interface control program (BASIC) listed in table 3.*

| Line number | Explanation |
|---|---|
| 1 | Sends the computer to the port set-up routines. |
| 2 | Sends the computer to the subroutine installing the real time clock. |
| 3 | Tells operator to turn on the switch which starts the pulse train. |
| 7–8 | Cause the computer to wait until the sample has been injected. |
| 10 | Sets up the array area to store 200 data points each for the switch-times, for GFAAS background intensities, for the GFAAS analyte signals, and for the signals from the UV detector. |
| 15–20 | Reset the real time clock to zero at the start of the run. (The ASCII value for zero is 48. The number 48 is stored in four consecutive video memory locations.) |
| 199–200 | Check whether the GFAAS door is open. |
| 205–206 | Set values for background and flag to zero. |
| 208–215 | Collect the background signals from the first five atomizations and calculate the average background signal. |
| 218–221 | Determine the switch-time by checking the appropriate locations in the video memory. |
| 225–240 | Turn on the appropriate bits in the port (memory location) that activate the injector relay and solenoid valve. The activated relay and solenoid valve place the sample and coanalyte valves in the inject position. |
| 240–265 | Initiate the GFAAS analysis sequence and activate the solenoid valve that places the sample and coanalyte valves in the load position. |
| 268–270 | Cause the computer to wait until the 'start the recorder signal' is received from the GFAAS indicating the beginning of the atomization cycle. |
| 271–272 | Cause the computer to go to the data acquisition subroutine at line 500. |
| 500–510 | Select from the four possible analogue inputs the input from the GFAAS. |
| 520 | Starts the analogue-to-digital conversion. |
| 524–500 | Set up a counter and then acquire 100 data points (1 point approximately every 100 milliseconds) during the 10 second atomization. These 100 intensity values are summed into the variable A, representing the area under the GFAAS peak. |
| 560 | Resets the above counter to 0. |
| 570–590 | Select the UV analogue input, perform the analogue-to-digital conversion, and store the result as a variable U. |
| 599–600 | Display the values of the switch time, the variable A (area under the GFAAS peak), the average background, and the background-corrected variable A (A minus 100 times the average background) on the CRT screen. Multiplication of the average background by 100 is necessary because the GFAAS signal is the sum of 100 values. |
| 610 | Returns the computer from the analogue-to-digital conversion subroutine to line 273. |
| 273–276 | Select the temperature analog input and monitor the temperature of the graphite cuvette measured by a thermocouple. Cooling by the nitrogen stream is delayed until the cuvette has cooled to approximately 750°C to avoid the possibility of shocking the cuvette with cold nitrogen. When the cuvette temperature has reached 750°C, the GFAAS is reset, the furnace door opened, and the cuvette cooled by a nitrogen stream. |
| 279–287 | Monitor the cuvette temperature until the cuvette has cooled sufficiently for another analysis sequence to begin. Because this temperature is much more critical than the temperature monitored in line 272–276 the average of five values is obtained to ensure that a spurious signal does not lead to premature injection of the next sample. |
| 300 | Returns the computer to line 200 to repeat the analysis cycle. |
| 999–1120 | This subroutine configures the peripheral interface adapters. |
| 18080–19011 | This subroutine reads the data statements and places them into the proper memory locations. The values in the data statements are the decimal equivalents of the assembly language program (table 1). By installing the program in this manner the assembler does not have to be installed and the assembly program loaded every time power is removed from the computer. |

arsenite solutions prepared from arsenic trichloride and distilled water a cuvette temperature of 150 °C at the time of injection was found to be optimal (figure 6). Similar shaped absorbance/temperature curves were found for copper [14], lead [15], and cadmium [16]. With injection at 150 °C and cooling of the cuvette with nitrogen, the time between analyses was reduced to approximately 50 s.

### Adaptation of the HPLC-GFAAS (IL-555) system to other graphite furnace atomic absorption spectrometers

The HPLC-GFAAS system described for the Instrument Laboratory Model IL-555 graphite furnace atomic absorption spectrometer can be used with other spectrometers after minor modification. The injection device might have to be adjusted to fit the geometry of the furnace. If the furnace does not have a door, the microswitch must be eliminated and line 200 in the BASIC program (table 3) must be replaced by a remark statement. In the IL-555 system the 'start the record signal' (+5 V) informs the computer that atomization begins. If other spectrometers do not have this signal, another signal must be used to notify the computer of the start of atomization. Such a signal might come from the atomization LED via a photocell or a direct connection. If the furnace does not have a thermocouple, lines 273–275 and 279–287 must be replaced by delay loops to allow the cuvette to cool sufficiently.

The Ohio Scientific Superboard II computer may be replaced by any 6502-based microprocessor (for example Apple II, Kim). The assembly language addresses (table 2) and line 18090 in the BASIC program (table 3) will have to be modified to be compatible with the chosen 6502-based computer. When a not-6502-based microprocessor is used, the assembly language program (table 2) and the real time clock subroutine in the BASIC program (table 3) must be rewritten. If the memory addresses used in the electronic interface are not available, the address buffering and decoding logic (figure 4) must be modified to decode an available memory address.

The hardcopy of the date line 599–600 (BASIC program, table 3) will have to be modified to correspond to the syntax of the computer. For graphical output a printer-specific subroutine will have to be written.

### Acknowledgements

Financial support for this project by the Robert A. Welch Foundation of Houston, Texas, and Texas A & M University's Center for Energy and Mineral Resources is gratefully acknowledged.

### References

1. Mertz, W., *Science*, **213** (1981), 1332.
2. Brinckman, F. E. and Bellama, J. M. (Eds), Organometals and organometalloids: occurrence and fate in the environment, *ACS Symposium Series, 82* (1978).
3. Brinckman, R. E., Blair, W. R., Jewett, K. L. and Iverson, W. P., *Journal of Chromatographic Science*, **15** (1977), 493.
4. Koizumi, H., Hadeishi, T. and McLaughlin, R., *Analytical Chemistry*, **50** (1978), 1700.
5. Stockton, R. A. and Irgolic, K. J., *International Journal of Environmental Analytical Chemistry*, **6** (1979), 313.
6. Irgolic, K. J., *Speciation of Arsenic Compounds in Water Supplies*, Report 1982, EPA/600/1–82/010, Order No. PB 82-2578717, 125 pp.; *Chemical Abstracts*, **98**, 113378 (1983).
7. Vickrey, T. M., Buren, M. S. and Howell, H. E., *Analytical Letters*, **A11** (1978), 1075.
8. Vickrey, T. M. and Eue, W., *Journal of Automatic Chemistry*, **1** (1979), 198.
9. Vickrey, T. M., Howell, H. E. and Paradise, M. T., *Analytical Chemistry*, **51** (1979), 1880.
10. Vickrey, T. M., Howell, H. E., Harrison, G. V. and Ramelow, G. J., *Analytical Chemistry*, **52** (1980), 1743.
11. Jinno, K., Tsuchida, S., Nakannishi, S., Hirata, Y. and Fujimoto, C., *Applied Spectroscopy*, **37** (1983), 258.
12. Irgolic, K. J., Stockton, R. A., Chakraborti, D. and Beyer, W., *Spectrochimica Acta*, **38B** (1983), 437.
13. Brinckman, F. E., Jewett, K. L., Iverson, W. P., Irgolic, K. J., Ehrdt, K.C. and Stockton, R. A., *Journal of Chromatography*, **191** (1980), 31.
14. Brown, L., Haswell, S. J., Rhead, M. M., O'Neill, P. and Bancroft, K. C. C., *Analyst*, **108** (1983), 1511.
15. Haswell, S. J., O'Neill, P. and Bancroft, K. C. C., Proc. Int. Conf. Heavy Metals in the Environment, Sept. 6–9, 1983, Heidelberg, Germany; published by CEP Consultants Ltd, 26 Albany Street, Edinburgh EH1 3QH, England. **2** (1983), 1215.
16. Haswell, S. J., Ph.D. Dissertation, Plymouth Polytechnic, Plymouth, England, 1983.

### NOTES FOR AUTHORS

*Journal of Automatic Chemistry incorporating Journal of Clinical Laboratory Automation* covers all aspects of automation and mechanization in analytical, clinical and industrial environments. The *Journal* publishes original research papers; short communications on innovations, techniques and instrumentation, or current research in progress; reports on recent commercial developments; and meeting reports, book reviews and information on forthcoming events. All research papers are refereed.

### Manuscripts

Two copies of articles should be submitted. All articles should be typed in double spacing with ample margins, on one side of the paper only. The following items should be sent: (1) a title-page including a brief and informative title, avoiding the word 'new' and its synonyms; a full list of authors with their affiliations and full addresses; (2) an abstract of about 250 words; (3) the main text; (4) appendices (if any); (5) references; (6) tables, each table on a separate sheet and accompanied by a caption; (7) illustrations (diagrams, drawings and photographs) numbered in a single sequence from 1 upwards and with the author's name on the back of every illustration; captions to illustrations should be typed on a separate sheet. Papers are accepted for publication on condition that they have been submitted only to this *Journal*.

### References

References should be indicated in the text by numbers following the author's name, i.e. Skeggs [6]. In the reference section they should be arranged thus:

to a journal

Manks, D. P., *Journal of Automatic Chemistry*, **3** (1981), 119.

to a book

Malmstadt, H. V., in *Topics in Automatic Chemistry*, Ed. Stockwell, P. B. and Foreman, J. K. (Horwood, Chichester, 1978), p. 68.

### Illustrations

Original copies of diagrams and drawings should be supplied, and should be drawn to be suitable for reduction to the page or column width of the *Journal*, i.e. to 85 mm or 179 mm, with special attention to lettering size. Photographs may be sent as glossy prints or as negatives.

### Proofs and offprints

The principal or corresponding author will be sent proofs for checking and will receive 50 offprints free of charge. Additional offprints may be ordered on a form which accompanies the proofs.

*Manuscripts should be sent to either Dr P. B. Stockwell or Ms M. R. Stewart, see inside front cover.*

